# Two ancient bacterial endosymbionts have coevolved with the planthoppers (Insecta: Hemiptera: Fulgoroidea)

**DOI:** 10.1186/1471-2148-12-87

**Published:** 2012-06-14

**Authors:** Julie M Urban, Jason R Cryan

**Affiliations:** 1 Nature Research Center, North Carolina Museum of Natural Sciences, Raleigh, NC, 27603, USA; 2 Research and Collections, North Carolina Museum of Natural Sciences, Raleigh, NC, 27601, USA

**Keywords:** Endosymbiont, Planthoppers, Fulgoroidea, *Vidania*, *Sulcia*, Codiversification

## Abstract

**Background:**

Members of the hemipteran suborder Auchenorrhyncha (commonly known as planthoppers, tree- and leafhoppers, spittlebugs, and cicadas) are unusual among insects known to harbor endosymbiotic bacteria in that they are associated with diverse assemblages of bacterial endosymbionts. Early light microscopic surveys of species representing the two major lineages of Auchenorrhyncha (the planthopper superfamily Fulgoroidea; and Cicadomorpha, comprising Membracoidea [tree- and leafhoppers], Cercopoidea [spittlebugs], and Cicadoidea [cicadas]), found that most examined species harbored at least two morphologically distinct bacterial endosymbionts, and some harbored as many as six. Recent investigations using molecular techniques have identified multiple obligate bacterial endosymbionts in Cicadomorpha; however, much less is known about endosymbionts of Fulgoroidea. In this study, we present the initial findings of an ongoing PCR-based survey (sequencing 16S rDNA) of planthopper-associated bacteria to document endosymbionts with a long-term history of codiversification with their fulgoroid hosts.

**Results:**

Results of PCR surveys and phylogenetic analyses of 16S rDNA recovered a monophyletic clade of Betaproteobacteria associated with planthoppers; this clade included *Vidania fulgoroideae*, a recently described bacterium identified in exemplars of the planthopper family Cixiidae. We surveyed 77 planthopper species representing 18 fulgoroid families, and detected *Vidania* in 40 species (representing 13 families). Further, we detected the *Sulcia* endosymbiont (identified as an obligate endosymbiont of Auchenorrhyncha in previous studies) in 30 of the 40 species harboring *Vidania*. Concordance of the *Vidania* phylogeny with the phylogeny of the planthopper hosts (reconstructed based on sequence data from five genes generated from the same insect specimens from which the bacterial sequences were obtained) was supported by statistical tests of codiversification. Codiversification tests also supported concordance of the *Sulcia* phylogeny with the phylogeny of the planthopper hosts, as well as concordance of planthopper-associated *Vidania* and *Sulcia* phylogenies.

**Conclusions:**

Our results indicate that the Betaproteobacterium *Vidania* is an ancient endosymbiont that infected the common ancestor of Fulgoroidea at least 130 million years ago. Comparison of our findings with the early light-microscopic surveys conducted by Müller suggests that *Vidania* is Müller’s *x*-symbiont, which he hypothesized to have codiversified with most lineages of planthoppers and with the *Sulcia* endosymbiont.

## Background

Many insect species in a diversity of hexapod orders are known to harbor obligate endosymbiotic bacteria [1-9]. Representing an extreme, members of the hemipteran suborder Auchenorrhyncha (commonly known as planthoppers, tree- and leafhoppers, spittlebugs, and cicadas [10]) exhibit an apparent “hunger for symbionts” [3]. Buchner’s [3] observations and the extensive light-microscopic surveys of his student, Müller [11-13], documented the presence of at least one symbiont in 395 of the 405 Auchenorrhyncha species examined; most host species harbored at least two morphologically distinct bacterial symbionts, and some harbored as many as six symbionts [3,13]. Müller also observed diversity in the morphology and location of the organs (bacteriomes) that house the endosymbionts within the insect hosts. Buchner [3] provided insight into the complexity of these endosymbiotic associations and arrangements in his summary of Müller’s work, as follows: “In order to present them and to understand them statistically, it is necessary to use letters to designate the forms and organs that proved to be different. Thus one speaks of *a*-, *b*-, *c*-, *d*-, *e*-symbionts and of *a*-organs, *b*-organs, and so forth. Sometimes there are so many that it is necessary to use Greek letters.” (p. 346). While Müller’s findings led Buchner to declare that Auchenorrhyncha is a “fairyland of symbioses” [3], the true diversity of endosymbiotic associations and arrangements in this insect lineage is likely even greater, as Müller surveyed less than 1% of the over 42,000 described species [14,15] of Auchenorrhyncha.

Auchenorrhyncha-bacteria symbioses remained virtually unexplored for the fifty years following Müller’s surveys. However, the development and widespread application of molecular techniques have reinvigorated the study of these endosymbiotic bacteria, which cannot be cultured outside of their hosts due to significant reductions in the bacterial genomes [9]. Using a PCR assay (i.e., amplification of the ribosomal gene 16S rDNA), Moran et al. [8] detected a bacterial endosymbiont in 30 Auchenorrhyncha species representing all four included superfamilies (Fulgoroidea, Membracoidea, Cercopoidea, and Cicadoidea, determined to be a monophyletic lineage within Hemiptera by Cryan & Urban [10]). That study detected a bacterium from the phylum Bacteroidetes in most Auchenorrhyncha surveyed, and the resulting bacterial phylogeny based on 16S sequences was concordant with the phylogeny of the host insects. Furthermore, observation of bacterial morphology using fluorescent in-situ hybridization (FISH) and localization of the endosymbiont to a bacteriome (examined in one spittlebug species and one leafhopper species) matched Müller’s description and illustrations of the “*a*-symbiont”. Consistent with Müller’s theory, Moran et al. [8] hypothesized that this endosymbiont, *Sulcia muelleri*, descended from a bacterium that infected a common ancestor of Auchenorrhyncha at least 260 million years ago, and through vertical transmission has codiversified with most Auchenorrhyncha lineages.

Several recent investigations identified additional obligate bacterial endosymbionts that co-occur with *Sulcia* in Cicadomorpha (one of the major monophyletic lineages within Auchenorrhyncha [14]) hosts: *Baumannia cicadellinicola* (Gammaproteobacteria) in leafhoppers (Membracoidea: Cicadellidae [16,17]; *Hodgkinia cicadicola* (Alphaproteobacteria) in cicadas (Cicadoidea: Cicadidae [18]), and *Zinderia insecticola* (Betaproteobacteria) in spittlebugs (Cercopoidea [19]). Genome sequencing of each of these pairs of endosymbionts (i.e., *Sulcia* + *BaumanniaSulcia* + *HodgkiniaSulcia* + *Zinderia*) revealed a pattern of genome reduction implicating the bacterial endosymbionts as nutrient provisioners to their insect hosts: genes that are retained in the bacterial genomes are those that encode essential nutrients absent in the host insects’ diet of plant sap [18-21]. These studies also demonstrated a remarkable convergence in that the genomes of the co-occurring endosymbiont pairs have co-evolved to be metabolically complementary [19]. For example, in leafhoppers, the endosymbiont *Sulcia* retains genes for the synthesis of eight (of ten) essential amino acids whereas *Baumannia* retains genes for the remaining two; in cicadas, *Sulcia* retains genes for eight amino acids whereas *Hodgkinia* retains genes for the remaining two; in spittlebugs, *Sulcia* retains genes for seven amino acids whereas *Zinderia* retains genes for the remaining three [19].

Less is known about the bacterial endosymbionts of Fulgoroidea (the other major lineage within Auchenorrhyncha [10]). Bressan et al. [22] identified the novel Gammaproteobacterium *Purcelliella pentastirinorum* in seven species of Cixiidae (including *Pentastiridius leporinus*, the vector of basses riches disease of beets). *Sulcia* was present in all seven of these planthopper species, and FISH observations showed that *Sulcia* and *Purcelliella* were segregated in separate bacteriomes within their host insects. Gonella et al. [23] identified the novel Betaproteobacterium *Vidania fulgoroideae*, along with *Sulcia*, in seven cixiid species (including *Hyalesthes obsoletus*, the vector of Bois noir grapevine disease); however, they were unable to localize *Vidania* to a bacteriome. Several studies investigated symbionts of various economically important planthopper species in the family Delphacidae (including the rice pests *Nilaparvata lugensSogatella furcifera*, and *Laodelphax striatellus*), documenting the presence of the bacterium *Wolbachia *[24,25] and a “yeast-like symbiont” [26,27]. *Sulcia* was not detected in those delphacid species, suggesting the loss of this obligate endosymbiont in some planthopper lineages, consistent with Müller [13] and Moran et al. [8].

Given these relatively few studies using molecular techniques, much remains to be learned about bacterial endosymbionts of Fulgoroidea (with more than 12,000 described species classified among ~20 families [28]). Müller’s [11-13] surveys of 217 species of Fulgoroidea documented a diversity of symbionts comparable to that found in Cicadomorpha hosts. Specifically, in addition to *Sulcia* (Müller’s *a*-symbiont), Müller observed another bacterium in many fulgoroid species that he termed the “*x*-symbiont”, hypothesizing that this bacterium infected the common ancestor of Fulgoroidea and codiversified with its hosts in most planthopper lineages. He also documented additional symbionts that he hypothesized were more recent associations that have codiversified with various lineages within the superfamily.

Here we present the initial findings of an ongoing PCR-based survey of planthopper-associated endosymbiotic bacteria to identify symbionts that have a long-term history of codiversification with various lineages of Fulgoroidea. We attempt to identify these endosymbionts by 1) surveying insect species representing most (18 of 21) recognized planthopper families, 2) performing phylogenetic reconstructions of the resulting bacterial DNA sequences, 3) comparing the bacterial phylogeny with the phylogeny of the planthopper hosts (reconstructed via a multi-locus phylogenetic approach based on sequence data generated from the same insect specimens from which the bacterial sequences were obtained), and 4) surveying the same insect taxa for the co-occurrence of *Sulcia* with any recovered endosymbiont.

## Results

### PCR screen and bacterial phylogeny

Initial screening of bacteria from a subset of the 77 sampled planthoppers yielded high quality 16S rDNA sequences. BLAST searches in GenBank showed highest similarity of these sequences to 16S sequences from diverse bacterial lineages in multiple phyla (Alphaproteobacteria, Betaproteobacteria, and Gammaproteobacteria). A preliminary phylogenetic reconstruction of these data (not shown) indicated that the sequences falling within the phylum Betaproteobacteria may be an endosymbiont because their recovered relationships were concordant with some well supported relationships within the planthopper phylogeny (based on [29]). We therefore focused on amplifying this Betaproteobacterial 16S from the remaining planthopper specimens. Sequences we obtained from bacteria falling within the Alphaproteobacteria and the Gammaproteobacteria are not presented here, as they are currently under further investigation to determine whether they represent additional planthopper-associated bacterial endosymbionts.

Betaproteobacterial 16S sequences were obtained from 40 of the 77 sampled planthopper species. Most of the sequences were approximately 1200–1400 bp long, although for some, only a shorter region of approximately 600–700 bp could be sequenced. Pairwise distances of planthopper Betaproteobacterial sequences averaged 25.1% (range, 0.3%-76.5%) under the maximum likelihood model of substitution specified by Modeltest results. The 16S sequence generated from the single exemplar of Derbidae was significantly divergent from the remaining planthopper sequences; pairwise distances excluding this sequence averaged 23.3% (range, 0.3%-54.3%).

To test for co-occurrence of *Sulcia* with the Betaproteobacterium, we attempted to amplify *Sulcia* 16S from the 40 planthoppers from which we sequenced the Betaproteobacterium; we obtained *Sulcia* sequences from 30 of these planthopper species. Most *Sulcia* 16S sequences were approximately 1300–1400 bp long (for several, only a shorter region of approximately 650–750 bp was obtained); pairwise distances of generated *Sulcia* sequences averaged 4.8% (range, 0%-11.2%).

**Figure 1:**
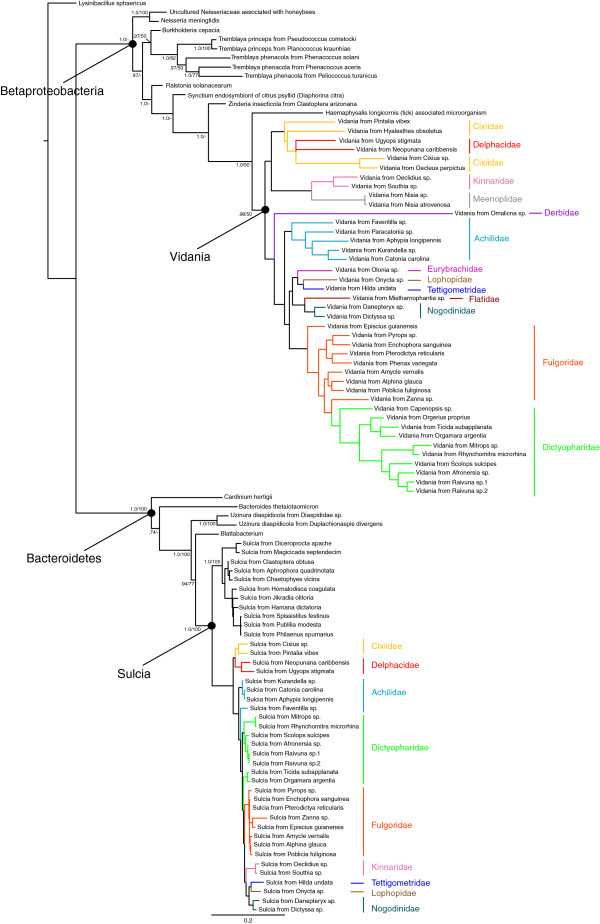
**ML topology of the complete bacteria data set. **BI posterior probability (pp) and ML bootstrap support (bs) values provided for nodes receiving support values greater than .50 pp or 50% bs. (Support values for relationships recovered within Fulgoroidea-associated *Vidania *and *Sulcia *clades given in Figures 2 and 3, respectively).

The topology resulting from the maximum likelihood (ML) analysis of the complete bacterial dataset (including all sequenced Betaproteobacterial and *Sulcia* 16S data) is shown in Figure 1; Bayesian Inference (BI) analysis yielded an almost identical topology (not shown). Betaproteobacteria and Bacteroidetes were each recovered as strongly supported monophyletic lineages. Within Betaproteobacteria, the clade associated with Fulgoroidea hosts was recovered as monophyletic, and included *Vidania* (sequenced from Cixiidae by Gonella et al. [23]) placed near sequences generated from exemplars of Cixiidae. Relationships recovered within this Fulgoroidea-associated Betaproteobacterial clade were concordant with the phylogeny of their insect hosts [29], including the recovery of several monophyletic lineages: Cixiidae + Delphacidae, Kinnaridae + Meenoplidae, and Fulgoridae + Dictyopharidae.

Within Bacteroidetes, the Auchenorrhyncha-associated *Sulcia* were recovered as a strongly supported monophyletic clade, within which was a monophyletic Fulgoroidea-associated *Sulcia* lineage. Relationships among the Fulgoroidea-associated *Sulcia* were generally concordant with the phylogeny of their planthopper hosts; disagreements in some placements are unsurprising given the low degree of *Sulcia* sequence variation as evidenced by many exceedingly short branches (Figure 1). Although previous studies [8,13] suggested the loss of *Sulcia* in some species of Delphacidae, we detected this endosymbiont in two (of 19) species surveyed (those two species were not included in the surveys of Müller [11-13] or Moran et al. [8]).

### Planthopper phylogeny

The 18S and 28S rDNA genes were amplified in three contiguous, overlapping fragments of approximately 600–700 bp each. The protein coding genes histone 3 (H3), wingless (Wg), and Cytochrome Oxidase I (COI) were each amplified as single fragments, with approximate lengths of 360 bp, 350 bp, and 900 bp, respectively. After ambiguously aligned regions of 18S and 28S were excluded, a combined dataset of approximately 5000 bp was obtained for each of the 40 Fulgoroidea exemplars from which the Betaproteobacterial endosymbionts were sequenced.

**Figure 2:**
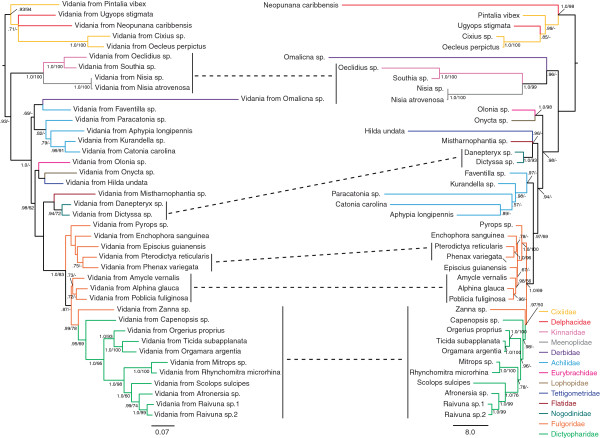
**ML topology of Fulgoroidea-associated *Vidania *(left) and ML topology of planthopper hosts from which *Vidania *was sequenced (right). **BI posterior probability (pp) and ML bootstrap support (bs) values provided for nodes receiving support values greater than .50 pp or 50% bs.

**Figure 3:**
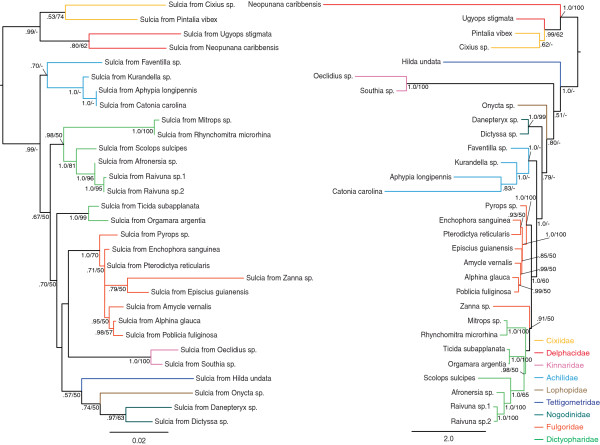
**ML topology of Fulgoroidea-associated *Sulcia *(left) and ML topology of planthopper hosts from which*Vidania *was sequenced (right). **BI posterior probability (pp) and ML bootstrap support (bs) values provided for nodes receiving support values greater than .50 pp or 50% bs.

Phylogenetic analyses of these Fulgoroidea species via both partitioned ML analysis and mixed-model BI analysis resulted in a single topology (Figures 2 and 3), which was consistent with previous reconstructions [29,30] in several aspects: the recovery of the monophyletic lineages Cixiidae + Delphacidae, Kinnaridae + Meenoplidae, Eurybrachidae + Lophopidae, and Fulgoridae + Dictyopharidae (including the fulgorid genus *Zanna* being placed with Dictyopharidae). However, the relative branching order of planthopper families was somewhat unusual in that the families Eurybrachidae, Lophopidae, Tettigometridae, Flatidae, and Nogodinidae were placed at more intermediate levels of the topology, relative to the positions of Fulgoridae and Dictyopharidae. Because we suspected that this result might represent an artifact of the limited taxonomic sampling in the present dataset, which we suspected could influence results of codiversification tests, unpartitioned ML searches were performed (as described below in Methods). These results yielded a topology more consistent with Urban & Cryan [29] in that the families Eurybrachidae, Lophopidae, Tettigometridae, Flatidae, and Nogodinidae were recovered as monophyletic and placed sister to Fulgoridae + Dictyopharidae. Therefore, two sets of codiversification tests were conducted (each comparing the planthopper Betaproteobacterial and the host phylogenies), using the two alternative host planthopper phylogenies (i.e., that based on the partitioned ML/BI analyses and that based on the unpartitioned ML analysis).

Phylogenetic reconstructions of the 30 exemplars of Fulgoroidea from which *Sulcia* was obtained showed the same pattern described above concerning results obtained using partitioned ML/BI analyses versus unpartitioned ML analysis. Therefore, two sets of codiversification tests between *Sulcia* and host phylogenies were conducted using the two alternative host planthopper phylogenies.

### Tests of codiversification

Tests of codiversification between the Fulgoroidea-associated Betaproteobacterial phylogeny and each of the two alternative insect-host phylogenies were conducted using TreeMap and ParaFit. Results obtained using TreeMap significantly rejected similarity between the bacterial and host trees due to chance alone when the partitioned ML/BI host tree was tested (p < 0.001) and when the unpartitioned host tree was tested (p < 0.001). Results obtained using ParaFit significantly rejected the global null hypothesis that the two trees are similar due to chance alone when the partitioned ML/BI host tree was tested (p = 0.001) and when the unpartitioned host tree was tested (p = 0.001).

Tests of codiversification comparing the Fulgoroidea-associated *Sulcia* phylogeny and each of the two alternative insect-host phylogenies using TreeMap significantly rejected similarity due to chance alone when the partitioned ML/BI host tree was tested (p < 0.001) and when the unpartitioned host tree was tested (p < 0.005). Results obtained using ParaFit significantly rejected the global null hypothesis that the two trees are similar due to chance alone when the partitioned ML/BI host tree was tested (p = 0.003) and when the unpartitioned host tree was tested (p = 0.004).

Tests of codiversification were also conducted comparing the planthopper *Sulcia* and Betaproteobacterial phylogenies for the 30 taxa from which both were amplifed. TreeMap results significantly rejected similarity due to chance alone (p < 0.001) as did ParaFit results (p = 0.001).

## Discussion

### Two ancient bacterial endosymbionts have coevolved with the planthoppers

Our results support the hypothesis that the Betaproteobacterium *Vidania*, first described by Gonella et al. [23] from four species of Cixiidae, is an ancient bacterial endosymbiont that infected the common ancestor of the superfamily Fulgoroidea and has since codiversified with its host insects (in most planthopper lineages) and with the co-occurring bacterial endosymbiont *Sulcia*. This conclusion is based on 1) the detection of *Vidania* in 40 species sampled from 13 of the 18 planthopper families tested, 2) the concordance of the *Vidania* phylogeny with the phylogeny of their planthopper hosts as supported by statistical tests of codiversification, 3) the co-occurrence of *Sulcia* and *Vidania* in 30 of the 40 screened planthoppers, and 4) the concordance of the *Sulcia* phylogeny with the phylogeny of their planthopper hosts as supported by statistical tests of codiversification.

As suggested by the relative branch lengths of *Vidania* and *Sulcia* (Figure 1), the 16S rDNA of Fulgoroidea-associated *Vidania* is evolving at a faster rate than that of Fulgoroidea-associated *Sulcia*. Pairwise distances of Fulgoroidea-associated *Vidania* sequences averaged 25.1% (range, 0.3%-76.5%); removing the exemplar of Derbidae, recomputed pairwise distances averaged 23.3% (range, 0.3%-54.3%). Pairwise distances of Fulgoroidea-associated *Sulcia* sequences averaged 4.8% (range, 0%-11.2%). This corresponds to a rate of evolution in *Vidania* 16S that is 4.85 times faster than that of *Sulcia* (after removing the derbid outlier), comparable to that observed by Takiya et al. [17] in two Cicadellidae-associated endosymbionts (in leafhopper hosts, *Baumannia* 16S was estimated to be evolving 4.88 times faster than *Sulcia* 16S).

Interestingly, the *Vidania* clade was placed (with strong support) as sister to the tick-associated microorganism (in this case, the endosymbiont was detected in the tick species *Haemaphysalis longicornis*) rather than as sister to the Cercopoidea-associated endosymbiont *Zinderia insecticola*, suggesting independence of these two bacterial infections within Auchenorrhyncha. Furthermore, it appears that the bacterial lineage giving rise to the *Tremblaya* endosymbionts of mealybugs is rather distantly related to *Vidania*, as each is more closely related to a free-living bacterium than to each other.

Moran et al. [8] surveyed for the presence of *Sulcia* in exemplars of five planthopper families, detecting the bacteria in two (Dictyopharidae and Fulgoridae); we detected *Sulcia* in exemplars of seven additional families of Fulgoroidea (Cixiidae, Delphacidae, Achilidae, Kinnaridae, Nogodinidae, Lophopidae, and Tettigometridae). Regarding *Sulcia* associations within the family Delphacidae, our findings are largely consistent with Müller’s [11-13] hypothesis that this endosymbiont was present in the more ancient delphacid lineages but was subsequently lost in younger delphacid lineages. The two delphacid species in which we detected *Sulcia* are from the subfamily Ugyopinae, which is placed among the oldest lineages within the phylogeny of Delphacidae [31].

### Hypothesized age and function of the *Vidania* endosymbiont

The earliest planthoppers known in the fossil record, all representing extinct families, are from the Jurassic and Permian periods (approximately 200–255 million years ago [32-34]). The oldest known fossils for extant planthopper families (specifically representing Cixiidae and Achilidae) are ~130 million years old [32,34]; diversification of Cixiidae is hypothesized to have occurred as early as ~200 mya (Szwedo, personal communication). Based on these data, we hypothesize that the *Vidania* association with planthoppers is at least 130 million years old and may be as old as 200 million years.

Given that the majority of insect-associated, obligate bacterial endosymbionts play a role in provisioning nutrients to their hosts (including the Cicadomorpha-associated *SulciaBaumanniaHodgkinia*, and *Zinderia* endosymbionts [9,19]), we hypothesize that *Vidania* is similarly involved in synthesizing and provisioning nutrients absent in phloem sap, the diet of the majority of planthopper species [35]. However, differences in the physical positions of bacteriomes in Cicadomorpha and Fulgoroidea [3,11,12] suggest that Fulgoroidea-associated *Vidania* and *Sulcia* may not exhibit the extreme metabolic complementarity seen in Cicadomorpha-associated endosymbionts. That is, in Cicadomorpha, bacteriomes are bilaterally symmetrical and multiple endosymbionts co-occur in the same, or adjacent, bacteriomes. Subsequent molecular studies support these observations [16,21], and suggest that the metabolic complementarity observed between Cicadomorpha-associated *Sulcia* and the companion endosymbiont is made possible by their close physical proximity which enables cross-feeding of metabolites [21]. In Fulgoroidea, however, Müller [11,12] observed that the relative positions of bacteriomes was strikingly more complex and variable than in Cicadomorpha; in Fulgoroidea hosts, each endosymbiont is segregated in its own bacteriome (none co-occur in the same bacteriome), and these bacteriomes are not necessarily adjacent. In fact, he observed that the relative positions of these bacteriomes vary both among and within planthopper families. Given these positional differences, the genomes of Fulgoroidea-associated *Sulcia* and *Vidania* may prove to exhibit some degree of metabolic redundancy, presuming *Vidania* is involved in nutrient provisioning.

### Solving for *X*: Is *Vidania* Müller’s *x*-symbiont?

In addition to documenting the presence/absence of particular endosymbionts within exemplars of various planthopper families, Müller’s work [11,12] illustrated the morphology of the endosymbiotic bacteria and of the bacteriomes housing them. Reaching a definitive conclusion as to whether *Vidania* is in fact Müller’s *x*-symbiont will require application of FISH analyses to visualize *Vidania* morphology, localize *Vidania* to a bacteriome, and visualize the morphology of the bacteriome. Gonella et al. [23] were unable to localize *Vidania* to a bacteriome, and our FISH analyses are currently underway. Based on findings presented here, we hypothesize *Vidania* is Müller’s *x*-symbiont, given the consistency of our findings with those of Müller (as summarized in sTable 1). We detected *Vidania* in exemplars of 13 of 18 planthopper families surveyed, including most families in which this endosymbiont was observed by Müller, as well as in one additional family not tested by Müller. Consistent with Müller’s hypothesis for the *x*-symbiont, our phylogenetic reconstructions and tests of codiversification indicate that *Vidania* has codiversified with its planthopper hosts. Also consistent with Müller’s observations of the *x*-symbiont occurring with the *a*-symbiont, we observed *Vidania* to co-occur with *Sulcia*, with results of statistical tests supporting the hypothesis of codiversification between planthopper *Sulcia* and *Vidania*.

**Table 1:** **Comparison of Müller’s**[3-5]**findings with results of present study**

**Planthopper family**	***Sulcia* (*a*-symbiont) detected by Müller**	***Sulcia* (*a*-symbiont) detected in present study**	***Vidania* (*x*-symbiont) detected by Müller**	**Vidania (*x*-symbiont) detected in present study**
Acanaloniidae	O	O	O	O
Achilidae	X	X	X	X
Achilixiidae	/	/	/	/
Caliscelidae	X	O	X	O
Cixiidae	X	X	X	X
Delphacidae	X	X	X	X
Derbidae	X	O	X	X
Dictyopharidae	X	X	X	X
Eurybrachidae	O	O	O	X
Flatidae	O	O	O	X
Fulgoridae	X	X	X	X
Gengidae	/	/	/	/
Hypochthonellidae	/	/	/	/
Issidae	X	O	X	O
Kinnaridae	/	X	/	X
Lophopidae	X	X	X	X
Meenoplidae	O	O	X	X
Nogodinidae	X	X	X	X
Ricaniidae	O	O	O	O
Tettigometridae	X	X	X	X
Tropiduchidae	O	O	X	O

X = endosymbiont detected; O = endosymbiont not detected; / = family not tested.

We were unable to detect *Vidania* in three families in which Müller detected the *x*-symbiont. Of the 77 planthoppers surveyed, we detected *Vidania* in 40, and of these, detected the co-occurrence of *Sulcia* in 30 taxa. Mapping of these presence/absence results onto the hypothesized phylogeny of Fulgoroidea (based on [29]) suggests a minimum of 11 losses of *Vidania* and 16 losses of *Sulcia* may have occurred within the diversification of the planthopper superfamily (shown in Supplemental Figure 1). However, we speculate that our results do not represent true endosymbiont losses, but instead, may be due to methodological difficulties associated either with quality/quantity of endosymbiont DNA present in the surveyed specimens (these were small specimens, and had been preserved in ethanol for approximately one year before endosymbiont DNA extraction was conducted), or with PCR amplification. Further investigation of these taxa using more recently collected specimens and more specific PCR primers is currently underway.

## Conclusions

Our results indicate that the Betaproteobacterium *Vidania* is an ancient endosymbiont that infected a common ancestor of the planthopper superfamily Fulgoroidea at least 130 million years ago, and since that time, has codiversified with most lineages of planthoppers and with the *Sulcia* endosymbiont. The consistency of these findings with the observations and hypotheses of Müller [11-13] suggests that *Vidania* is Müller’s *x*-symbiont; FISH analyses are currently underway to examine the morphology of *Vidania* to compare with Müller’s depictions of the *x*-symbiont’s morphology. Although the *Zinderia* endosymbiont of spittlebugs also arises from the phylum Betaproteobacteria, our findings suggest that these represent two independent bacterial infections within Auchenorrhyncha. The functional role(s) played by *Vidania* in the biology of its planthopper hosts may involve nutrient-provisioning, although this remains to be tested. Because planthoppers house their multiple bacterial endosymbionts differently than do other lineages of insects within Auchenorrhyncha, further study of planthopper endosymbioses promises to provide important insight into how bacterial symbioses are managed by the animals that serve as hosts.

## Methods

### DNA extraction and PCR screens for endosymbionts

The set of planthopper species screened for endosymbionts consisted of 77 species, selected from 18 of 21 recognized families of Fulgoroidea (Table 2). Insect specimens were collected into 95-100% ethanol and stored at −80°C in the New York State Museum’s Genome Bank (Albany, NY, USA). Endosymbiont DNA was extracted from the entire insect abdomen (or in the case of several large representatives of Fulgoridae, from half of the abdomen, sectioned longitudinally) using Qiagen DNEasy Kits (Qiagen, Inc., Valencia, CA, USA). The PCR primers used for initial screening for endosymbionts were a series of universal primers (Table 3). Sequences obtained from initial screening of some specimens were searched against GenBank and preliminary phylogenetic analyses were conducted (not published). Because these results suggested the presence of a Betaproteobacterial endosymbiont, the primers 8FBAC and 1492RBac used by Gruwell et al. [36] to amplify the endosymbiont *Tremblaya princeps* from mealybugs were used to more specifically target this Betaproteobacterium. The planthopper specimens from which the Betaproteobacterial endosymbiont 16S was successfully sequenced were subsequently screened for the *Sulcia* endosymbiont. The primers used to amplify *Sulcia* 16S were the Bacteroidetes-specific primers 10_CFB_FF and 1515_R from Moran et al. [8].

**Table 2:** Planthopper taxa screened for endosymbionts

**Taxon**	**Voucher code**	**Geographical source**	**Betaproteobacterium detected**
**Acanaloniidae**			
*Acanalonia bivitatta*	02-06-17-81	USA: New York	--
*Acanalonia fasciata*	10-01-01-14	USA: New Mexico	--
**Achilidae**			
*Aphypia longipennis*	09-07-25-09	Ghana	+
*Catonia carolina*	04-12-17-13	USA: Louisiana	+
*Faventilla* sp.	09-07-15-59	Malaysia	+
*Kurandella* sp.	10-01-07-07	Dominican Republic	+
*Paracatonia* sp.	09-07-25-31	Bolivia	+
**Caliscelidae**			
*Aphelonema* sp.	02-06-20-23	USA: Delaware	--
**Cixiidae**			
*Cixius* sp.	09-08-25-72	USA: Arizona	+
*Oecleus perpictus*	10-01-01-26	USA: Arizona	+
*Pintalia vibex*	02-06-20-08	USA: Delaware	+
*Benna* sp.	09-07-15-60	Malaysia	--
*Bothriocera daedala*	04-11-29-08	Dominica	--
*Oliarus placitus*	02-06-20-09	USA: Delaware	--
**Delphacidae**			
*Neopunana caribbensis*	01-07-24-65	USVI: St. John	+
*Ugyops stigmata*	02-09-11-51	Belize	+
*Burnilia* sp.	02-09-11-55	Belize	--
*Copicerus irroratus*	02-01-10-54	USA: Maryland	--
*Javesella pellucida*	01-07-24-11	USA: Pennsylvania	--
*Megamelus distinctus*	01-07-24-09	USA: North Carolina	--
*Nilaparvata serratta*	04-12-09-06	Costa Rica	--
*Nilaparvata wolcotti*	DEL043	USA: Utah	--
*Opiconsiva* sp.	04-12-09-67	Australia	--
*Peregrinus maidis*	01-07-24-22	USA: Delaware	--
*Saccharosydne saccharivora*	01-07-24-06	USA: Florida	--
*Sogatella kolophon*	04-12-09-68	Australia	--
*Stenocranus* sp.1	01-07-24-01	USA: Utah	--
*Stenocranus* sp.2	01-07-24-17	USA: Utah	--
*Stenocranus* sp.3	02-01-10-68	USA: Delaware	--
*Stobaera concinna*	01-07-24-12	USA: Florida	--
*Tagosodes pusanus*	04-12-09-81	Australia	--
*Tropidocephala* sp.	03-02-01-15	Papua New Guinea	--
*Ugyops* sp.	03-02-01-16	Papua New Guinea	--
**Derbidae**			
*Omalicna* sp.	04-12-17-14	USA: Florida	+
*Anotia binnetii*	10-01-05-77	USA: Florida	--
*Anotia westwoodii*	02-06-20-07	USA: Tennessee	--
*Cedusa obscura*	02-06-17-06	USA: New York	--
**Dictyopharidae**			
*Afronersia* sp.	09-08-01-75	Zambia	+
*Capenopsis* sp.	09-08-11-01	South Africa	+
*Mitrops* sp.	09-08-15-01	Argentina	+
*Orgamara argentia*	10-01-01-08	USA: Arizona	+
*Orgerius proprius*	09-08-25-48	USA: California	+
*Raivuna* sp.1	04-12-28-51	India	+
*Raivuna* sp.2	04-12-28-52	India	+
*Rhynchomitra microrhina*	02-06-20-22	USA: North Carolina	+
*Scolops sulcipes*	02-06-17-07	USA: New York	+
*Ticida subapplanata*	09-08-25-63	USA: California	+
*Hyalodictyon* sp.	04-12-17-57	French Guiana	--
*Lappida* sp.	03-07-21-58	Costa Rica	--
**Eurybrachidae**			
*Olonia* sp.	04-12-28-11	Australia	+
*Platybrachys* sp.	03-07-21-81	Australia	--
**Flatidae**			
*Mistharnophantia* sp.	04-12-17-33	USA: Arizona	+
*Metcalfa pruinosa*	03-07-21-43	USA: New York	--
**Fulgoridae**			
*Alphina glauca*	03-01-09-76	USA: New Mexico	+
*Amycle vernalis*	03-07-21-71	USA: South Carolina	+
*Enchophora sanguinea*	04-06-25-16	Costa Rica	+
*Episcius guianensis*	04-12-17-81	French Guiana	+
*Phenax variegata*	04-10-30-18	Peru	+
*Poblicia fuliginosa*	03-01-09-51	USA: Louisiana	+
*Pterodictya reticularis*	04-05-15-79	Costa Rica	+
*Pyrops* sp.	04-12-15-30	India	+
*Zanna* sp.1	05-02-10-32	Zambia	+
*Penthicodes pulchella*	04-12-15-34	India	--
*Zanna* sp.2	04-12-28-80	Ghana	--
**Issidae**			
*Thionia argo*	02-06-17-10	USVI: St. John	--
**Kinnaridae**			
*Oeclidius* sp.	03-07-21-72	USA: California	+
*Southia* sp.	10-01-05-10	Dominican Republic	+
**Lophopidae**			
*Onycta* sp.	03-01-09-55	Papua New Guinea	+
*Lophops* sp.	03-01-09-60	Papua New Guinea	--
**Meenoplidae**			
*Nisia atrovenosa*	09-08-25-08	France	+
*Nisia* sp.	03-01-09-65	Papua New Guinea	+
**Nogodinidae**			
*Danepteryx* sp.	04-12-17-23	USA: California	+
*Dictyssa* sp.	04-12-17-39	USA: California	+
**Ricaniidae**			
*Euricania* sp.	02-06-17-25	Papua New Guinea	--
*Pochazia guttifera*	02-06-17-34	Papua New Guinea	--
**Tettigometridae**			
*Hilda undata*	04-12-28-72	Ghana	+
**Tropiduchidae**			
*Tangia viridis*	02-06-17-09	USVI: St. John	--

**Table 3:** Primers used for endosymbiont PCR amplifications

**Primer name**	**Description**	**Sequence**	**Reference**
10 F*	Universal bacteria	AGT TTG ATC ATG GCT CAG ATT G	Moran et al., 2003
1507R	Universal bacteria	TAC CTT GTT ACG ACT TCA CCC CAG	Moran et al., 2003
35R	Universal bacteria	CCT TCA TCG CCT CTG ACT GC	Takiya et al., 2006
8FBAC*	Universal bacteria	AGA GTT TGA TCC TGG CTC AG	Gruwell et al., 2010
1492RBac	Universal bacteria	GGT TAC CTT GTT ACG ACT T	Gruwell et al., 2010
640 F*	Universal bacteria	GGT GTA GCG GTG AAA TGC	newly designed
720 F*	Universal bacteria	GGA TTA GAT ACC CTG GTA GTC C	newly designed
740R	Universal bacteria	GGA CTA CCA GGG TAT CTA ATC C	newly designed
10 CFB FF*	*Sulcia*	AGA GTT TGA TCA TGG CTC AGG ATG	Moran et al., 2005
1515R	*Sulcia*	GTA CGG CTA CCT TGT TAC GAC TTA G	Moran et al., 2005

Forward primers denoted by *.

Oligonucleotide primers used in PCR reactions were synthesized by Integrated DNA Technologies, Inc. (Coralville, IA, USA). Betaproteobacterial endosymbiont 16S was amplified in 25 μl reactions using Qiagen DNA polymerase (Qiagen, Inc.) under the following cycling protocol: 3 min. “hot start” at 94°C, 30–35 cycles of 1 min. at 51-55°C and 1:30 min. at 72°C, with final extension at 72°C for 10 min. *Sulcia* 16S was amplified in 25 μl reactions using Qiagen DNA polymerase and the following thermal cycler protocol was used: 3 min. “hot start” at 94°C, 30–35 cycles of 1 min. at 51-58°C and 1 min. at 72°C, with final extension at 72°C for 10 min. Each PCR was run with negative controls. Amplified DNA was visualized using 1-2% agarose gel electrophoresis with ethidium-bromide staining. DNA products were purified using ExoSAPIT (GE Healthcare, Piscataway, NJ, USA). Purified PCR products were sent to the Center for Functional Genomic Laboratory at the University at Albany (Albany, NY, USA) for sequencing (using the same primers as used in PCR amplification).

### DNA extraction and PCR amplification of planthopper host sequences

Nucleotide sequence data were generated from five gene regions of the host planthoppers from which the Betaproteobacterial endosymbiont was successfully amplified. Insect DNA was typically extracted from either thoracic or leg muscle tissue using Qiagen DNEasy Kits (Qiagen, Inc., Valencia, CA, USA). For some specimens, insect DNA was obtained through the whole abdomen extraction describe above (as used for obtaining endosymbiont DNA). The five gene regions that were amplified and sequenced were the nuclear ribosomal genes 18S and 28S rDNA, the nuclear protein coding genes histone H3 (H3) and wingless (Wg), and the mitochondrial protein coding gene cytochrome oxidase I (COI). All planthopper host data were generated by the authors of the present study, with some sequences newly generated and others published in previous studies [29-31]. Newly generated sequences were obtained following the protocols described in Urban et al. [31]. All planthopper host data (newly generated sequences and those previously published) were obtained from the same specimen from which the corresponding endosymbiont data were generated.

### Endosymbiont sequences and bacterial alignment

All chromatographic data were inspected visually, assembled into contiguous sequences, and edited using Sequencher 4.10.1 for Windows (GeneCodes, 2010). Outgroup bacterial 16S sequences were obtained from GenBank and included in the alignment with the endosymbiont data (Table 4). These included representatives of other insect endosymbionts and free-living bacteria from diverse lineages within the Bacteroidetes and Betaproteobacteria. Bacterial 16S sequences were aligned according to secondary structure using the Ribosomal Database Project, Release 10 [37]. Ambiguous regions of the alignment (seven regions with a combined length of 352 bp) were visually identified and excluded from all phylogenetic analyses.

**Table 4:** Bacterial sequences

**Bacteria name**	**Host (if applicable)**	**Host family**	**GenBank number**	**Host voucher code**
Phylum Betaproteobacteria				
*Candidatus* Vidania fulgoroideae	*Aphypia longipennis*	Achilidae	JQ982542	ACH015
*Candidatus* Vidania fulgoroideae	*Faventilla* sp.	Achilidae	JQ982543	ACH016
*Candidatus* Vidania fulgoroideae	Catonia carolina*	Achilidae	JQ982544	ACH017
*Candidatus* Vidania fulgoroideae	*Kurandella* sp.	Achilidae	JQ982545	ACH018
*Candidatus* Vidania fulgoroideae	*Paracatonia* sp.	Achilidae	JQ982546	ACH019
*Candidatus* Vidania fulgoroideae	Pintalia vibex*	Cixiidae	JQ982547	CIX024
*Candidatus* Vidania fulgoroideae	*Cixius* sp.	Cixiidae	JQ982548	CIX028
*Candidatus* Vidania fulgoroideae	*Oecleus perpictus*	Cixiidae	JQ982549	CIX029
*Candidatus* Vidania fulgoroideae	*Neopunana caribbensis*	Delphacidae	JQ982550	DEL081
*Candidatus* Vidania fulgoroideae	Ugyops stigmata*	Delphacidae	JQ982551	DEL131
*Candidatus* Vidania fulgoroideae	*Omalicna* sp.	Derbidae	JQ982552	DER006
*Candidatus* Vidania fulgoroideae	Scolops sulcipes*	Dictyopharidae	JQ982553	DIC001
*Candidatus* Vidania fulgoroideae	Rhynchomitra microrhina*	Dictyopharidae	JQ982554	DIC002
*Candidatus* Vidania fulgoroideae	*Raivuna* sp.1	Dictyopharidae	JQ982555	DIC007
*Candidatus* Vidania fulgoroideae	*Raivuna* sp.2	Dictyopharidae	JQ982556	DIC008
*Candidatus* Vidania fulgoroideae	*Afronersia* sp. *	Dictyopharidae	JQ982557	DIC035
*Candidatus* Vidania fulgoroideae	*Capenopsis* sp.	Dictyopharidae	JQ982558	DIC036
*Candidatus* Vidania fulgoroideae	*Mitrops* sp.	Dictyopharidae	JQ982559	DIC063
*Candidatus* Vidania fulgoroideae	*Orgeriu proprius*	Dictyopharidae	JQ982560	DIC094
*Candidatus* Vidania fulgoroideae	*Ticida subapplanata*	Dictyopharidae	JQ982561	DIC103
*Candidatus* Vidania fulgoroideae	*Orgamara argentia*	Dictyopharidae	JQ982562	DIC112
*Candidatus* Vidania fulgoroideae	*Olonia* sp.*	Eurybrachidae	JQ982563	EUR004
*Candidatus* Vidania fulgoroideae	*Mistharnophantia* sp.*	Flatidae	JQ982564	FLA019
*Candidatus* Vidania fulgoroideae	Poblicia fuliginosa*	Fulgoridae	JQ982565	FUL004
*Candidatus* Vidania fulgoroideae	Alphina glauca*	Fulgoridae	JQ982566	FUL009
*Candidatus* Vidania fulgoroideae	Amycle vernalis*	Fulgoridae	JQ982567	FUL011
*Candidatus* Vidania fulgoroideae	Pterodictya reticularis*	Fulgoridae	JQ982568	FUL021
*Candidatus* Vidania fulgoroideae	Phenax variegata*	Fulgoridae	JQ982573	FUL073
*Candidatus* Vidania fulgoroideae	Enchophora sanguinea*	Fulgoridae	JQ982569	FUL031
*Candidatus* Vidania fulgoroideae	*Pyrops* sp.	Fulgoridae	JQ982570	FUL048
*Candidatus* Vidania fulgoroideae	Episcius guianensis*	Fulgoridae	JQ982571	FUL053
*Candidatus* Vidania fulgoroideae	*Zanna* sp.	Fulgoridae	JQ982572	FUL065
*Candidatus* Vidania fulgoroideae	*Oeclidius* sp.*	Kinnaridae	JQ982576	KIN002
*Candidatus* Vidania fulgoroideae	*Southia* sp.	Kinnaridae	JQ982577	KIN004
*Candidatus* Vidania fulgoroideae	*Onycta* sp.*	Lophopidae	JQ982578	LOP003
*Candidatus* Vidania fulgoroideae	*Nisia* sp.	Meenoplidae	JQ982579	MEE001
*Candidatus* Vidania fulgoroideae	*Nisia atrovenosa*	Meenoplidae	JQ982580	MEE005
*Candidatus* Vidania fulgoroideae	*Danepteryx* sp.*	Nogodinidae	JQ982574	ISS013
*Candidatus* Vidania fulgoroideae	*Dictyssa* sp.	Nogodinidae	JQ982575	ISS014
*Candidatus* Vidania fulgoroideae	Hilda undata*	Tettigometridae	JQ982581	TET003
*Candidatus* Vidania fulgoroideae	*Hyalesthes obsoletus*	Cixiidae	FR686932	
*Candidatus* Tremblaya phenacola	*Peliococcus turanicus*	Pseudococcidae	HM449974	
*Candidatus* Tremblaya phenacola	*Phenacoccus aceris*	Pseudococcidae	HM449982	
*Candidatus* Tremblaya phenacola	*Phenacoccus solani*	Pseudococcidae	HM449980	
*Candidatus* Tremblaya princeps	*Planocococcus kraunhiae*	Pseudococcidae	AB374415	
*Candidatus* Tremblaya princeps	*Pseudococcus comstocki*	Pseudococcidae	AB374416	
*Candidatus* Zinderia insecticola	*Clastoptera arizonana*	Clastopteridae	CP002161	
*Haemaphysalis longicornis* (tick) associated microorganism	*Haemaphysalis longicornis*	Ixodidae	AB001520	
Synctium endosymbiont of citrus psyllid	*Diaphorina citra*	Psyllidae	AB038368	
Uncultured Neisseriaceae associated with honeybees	*Bombus sonorus*	Apidae	HM108668	
*Burkholderia cepacia*	(free-living)		AY512825	
*Neisseria meningtidis*	(free-living)		GQ294480	
*Ralstonia solanacearum*	(free-living)		AY712685	
Phylum Bacteroidetes				
*Candidatus* Sulcia muelleri	*Aphypia longipennis*	Achilidae	JQ982613	ACH015
*Candidatus* Sulcia muelleri	*Faventilla* sp.	Achilidae	JQ982614	ACH016
*Candidatus* Sulcia muelleri	*Catonia carolina*	Achilidae	JQ982615	ACH017
*Candidatus* Sulcia muelleri	*Kurandella* sp.	Achilidae	JQ982616	ACH018
*Candidatus* Sulcia muelleri	*Pintalia vibex*	Cixiidae	JQ982617	CIX024
*Candidatus* Sulcia muelleri	*Cixius* sp.*	Cixiidae	JQ982618	CIX028
*Candidatus* Sulcia muelleri	Neopunana caribbensis*	Delphacidae	JQ982619	DEL081
*Candidatus* Sulcia muelleri	Ugyops stigmata*	Delphacidae	JQ982620	DEL131
*Candidatus* Sulcia muelleri	*Scolops sulcipes*	Dictyopharidae	EU646046	DIC001
*Candidatus* Sulcia muelleri	*Rhynchomitra microrhina*	Dictyopharidae	JQ982621	DIC002
*Candidatus* Sulcia muelleri	*Raivuna* sp.1	Dictyopharidae	JQ982622	DIC007
*Candidatus* Sulcia muelleri	*Raivuna* sp.2	Dictyopharidae	JQ982623	DIC008
*Candidatus* Sulcia muelleri	*Afronersia* sp.	Dictyopharidae	JQ982624	DIC035
*Candidatus* Sulcia muelleri	*Mitrops* sp.	Dictyopharidae	JQ982625	DIC063
*Candidatus* Sulcia muelleri	*Ticida subapplanata*	Dictyopharidae	JQ982626	DIC103
*Candidatus* Sulcia muelleri	*Orgamara argentia*	Dictyopharidae	JQ982627	DIC112
*Candidatus* Sulcia muelleri	*Poblicia fuliginosa*	Fulgoridae	EU646053	FUL004
*Candidatus* Sulcia muelleri	*Alphina glauca*	Fulgoridae	EU646055	FUL009
*Candidatus* Sulcia muelleri	*Amycle vernalis*	Fulgoridae	EU646057	FUL011
*Candidatus* Sulcia muelleri	*Pterodictya reticularis*	Fulgoridae	JQ982628	FUL021
*Candidatus* Sulcia muelleri	*Enchophora sanguinea*	Fulgoridae	EU646065	FUL031
*Candidatus* Sulcia muelleri	*Pyrops* sp.	Fulgoridae	JQ982629	FUL048
*Candidatus* Sulcia muelleri	*Episcius guianensis*	Fulgoridae	EU646077	FUL053
*Candidatus* Sulcia muelleri	*Zanna* sp.	Fulgoridae	JQ982630	FUL065
*Candidatus* Sulcia muelleri	*Oeclidius* sp.	Kinnaridae	JQ982633	KIN002
*Candidatus* Sulcia muelleri	*Southia* sp.	Kinnaridae	JQ982634	KIN004
*Candidatus* Sulcia muelleri	*Onycta* sp.	Lophopidae	JQ982635	LOP003
*Candidatus* Sulcia muelleri	*Danepteryx* sp.	Nogodinidae	JQ982631	ISS013
*Candidatus* Sulcia muelleri	*Dictyssa* sp.	Nogodinidae	JQ982632	ISS014
*Candidatus* Sulcia muelleri	*Hilda undata*	Tettigometridae	JQ982636	TET003
*Candidatus* Sulcia muelleri	*Aphrophora quadrinotata*	Aphrophoridae	DQ066629	
*Candidatus* Sulcia muelleri	*Philaenus spumarius*	Aphrophoridae	DQ066636	
*Candidatus* Sulcia muelleri	*Hamana dictatoria*	Cicadellidae	DQ066638	
*Candidatus* Sulcia muelleri	*Homalodisca coagulata*	Cicadellidae	DQ066646	
*Candidatus* Sulcia muelleri	*Jikradia olitoria*	Cicadellidae	DQ066640	
*Candidatus* Sulcia muelleri	*Diceroprocta apache*	Cicadidae	DQ066626	
*Candidatus* Sulcia muelleri	*Magicicada septendecim*	Cicadidae	DQ066625	
*Candidatus* Sulcia muelleri	*Clastoptera obtusa*	Clastopteridae	DQ066634	
*Candidatus* Sulcia muelleri	*Chaetophyes vicina*	Machaerotidae	DQ066633	
*Candidatus* Sulcia muelleri	*Publilia modesta*	Membracidae	DQ066641	
*Candidatus* Sulcia muelleri	*Spissistilus festinus*	Membracidae	DQ066637	
*Blattabacterium*	*Cryptocercus* sp.	Cryptocercidae	Z35664	
*Candidatus* Uzinura diaspidicola	*Duplachionaspis divergens*	Diaspididae	DQ868825	
*Candidatus* Uzinura diaspidicola	Diaspididae sp. MD0002B	Diaspididae	GQ424953	
*Candidatus* Cardinium hertigii	*Encarsia hispida*	Aphelinidae	AY331187	
*Bacteroides thetaiotaomicron*	(human intestinal gut microbe)		NC004663	
Phylum Firmicutes (outgroup root)				
*Lysinibacillus sphaericus*	(free-living)		FJ528593	

* Indicates that shorter (~600-750 bp) regions of 16S were amplified for these taxa.

Note: Host voucher code provided only for surveyed planthopper specimens from which new endosymbiont data were generated.

### Planthopper host sequences and alignment

All chromatographic data were inspected visually, assembled into contiguous sequences, and edited using Sequencher 4.10.1 for Windows (GeneCodes, 2010). Multiple sequence alignments for 18S and 28S data were initially performed manually, and were then improved upon using the sequence alignment program MAFFT 6 (online version), with the Q-INS-i iterative refinement algorithm [38]. Highly variable regions of 18S (one region of length 39 bp) and 28S (three regions of combined length 336 bp) were excluded from phylogenetic analysis because of extreme ambiguity in alignment. Multiple sequence alignments for H3 and COI were unambiguous and contained no gaps. The multiple sequence alignment for Wg contained one gap, but it did not interrupt or shift the reading frame. GenBank accession numbers for all planthopper sequences are provided in Table 5.

**Table 5:** Planthopper sequences used to reconstruct host phylogeny

**Taxon**	**Voucher code**	**18S**	**28S**	**H3**	**Wg**	**COI**
**Achilidae**						
*Aphypia longipennis*	ACH015	JQ982508	JQ982525	JQ982599	--	JQ982582
*Catonia carolina*	ACH017	JQ982510	JQ982527	JQ982600	--	JQ982584
*Faventilla* sp.	ACH016	JQ982509	JQ982526	--	--	JQ982583
*Kurandella* sp.	ACH018	JQ982511	JQ982528	JQ982601	--	JQ982585
*Paracatonia* sp.	ACH019	JQ982512	JQ982529	JQ982602	--	JQ982586
**Cixiidae**						
*Cixius* sp.	CIX028	JQ982514	JQ982531	JQ982604	--	--
**Delphacidae**						
*Oecleus perpictus*	CIX029	JQ982515	JQ982532	JQ982605	--	--
*Pintalia vibex*	CIX024	JQ982513	JQ982530	JQ982603	--	--
*Neopunana caribbensis*	DEL081	HM017276	HM017384	--	--	HM017495
*Ugyops stigmata*	DEL131	HM017301	HM017409	--	--	HM017501
**Derbidae**						
*Omalicna* sp.	DER006	DQ532519	DQ532599	DQ532673	DQ532739	--
**Dictyopharidae**						
*Afronersia* sp.	DIC035	JQ982516	JQ982533	JQ982606	JQ982637	JQ982587
*Capenopsis* sp.	DIC036	JQ982517	JQ982534	JQ982607	JQ982638	JQ982588
*Mitrops* sp.	DIC063	JQ982518	JQ982535	--	JQ982639	JQ982589
*Orgamara argentia*	DIC112	JQ982521	JQ982538	JQ982609	--	JQ982591
*Orgerius proprius*	DIC094	JQ982519	JQ982536	JQ982608	--	JQ982590
*Raivuna* sp.1	DIC007	DQ532526	DQ532606	DQ532679	DQ532744	EU645978
*Raivuna* sp.2	DIC008	EU645746	EU645810	EU645877	EU645926	EU645979
*Rhynchomitra microrhina*	DIC002	DQ532523	DQ532603	DQ532676	--	EU645975
*Scolops sulcipes*	DIC001	DQ532522	DQ532602	DQ532675	DQ532742	EU645974
*Ticida subapplanata*	DIC103	JQ982520	JQ982537	--	--	--
**Eurybrachidae**						
*Olonia* sp.	EUR004	DQ532531	DQ532611	DQ532684	DQ532748	JQ982592
**Flatidae**						
*Mistharnophantia* sp.	FLA019	JQ982522	JQ982539	JQ982610	--	--
**Fulgoridae**						
*Alphina glauca*	FUL009	EU645751	EU645818	EU645882	--	EU645987
*Amycle vernalis*	FUL011	EU645752	EU645819	EU645883	--	--
*Enchophora sanguinea*	FUL031	EU645769	EU645836	EU645898	JQ982640	EU646004
*Episcius guianensis*	FUL053	EU645789	EU645856	--	EU645957	EU646026
*Phenax variegata*	FUL073	EU645790	EU645857	--	EU645958	EU646027
*Poblicia fuliginosa*	FUL004	EU645748	EU645814	--	EU645929	EU645984
*Pterodictya reticularis*	FUL021	EU645761	EU645828	EU645891	EU645935	EU645997
*Pyrops* sp.	FUL048	EU645785	EU645852	EU645910	EU645953	EU646021
*Zanna* sp.	FUL065	EU645801	EU645868	JQ982611	EU645967	EU646038
**Kinnaridae**						
*Oeclidius* sp.	KIN002	DQ532551	DQ532631	DQ532703	DQ532765	JQ982594
*Southia* sp.	KIN004	JQ982523	JQ982540	--	--	JQ982595
**Lophopidae**						
*Onycta* sp.	LOP003	DQ532554	DQ532634	DQ532706	DQ532768	JQ982596
**Meenoplidae**						
*Nisia* sp.	MEE001	DQ532557	DQ532637	DQ532709	DQ532771	--
*Nisia atrovenosa*	MEE005	JQ982524	JQ982541	JQ982612	--	JQ982597
**Nogodinidae**						
*Danepteryx* sp.	ISS013	DQ532547	DQ532627	DQ532699	DQ532761	JQ982593
*Dictyssa* sp.	ISS014	DQ532548	DQ532628	DQ532700	DQ532762	--
**Tettigometridae**						
*Hilda undata*	TET003	DQ532567	DQ532647	DQ532719	DQ532779	JQ982598

### Phylogenetic reconstruction of bacterial data

Phylogenetic analyses of the bacterial dataset were conducted using Maximum Likelihood (ML) and Bayesian Inference (BI) reconstruction methodologies. Under both methods, gaps were treated as missing data. Modetest 3.7 [39] was used to determine the best-fitting model for the bacterial 16S data. Results of the Akaike information criterion (AIC [40]) indicated that the GTR + I + G model was the best-fitting model for these data. ML analysis was conducted on the bacterial data using GARLI 2.0 for Windows [41]. Ten independent search replicates were run under the GTR + I + G model, with each replicate run for 100,000 generations. Bootstrap support values for nodes on the ML topology were computed with GARLI by running 100 bootstrap replicates. Bayesian analysis of the bacterial data was conducted using MrBayes 3.1.2 [42] on the CIPRES Science Gateway [43]. The Bayesian analysis was run under the GTR + I + G model for 20 million generations. Two independent runs were performed, each with four chains (three heated and one cold), uninformative priors, and trees sampled at intervals of 1000 generations. Stationarity was determined by examining log-likelihood scores plotted across generations with Tracer [44] and by examining standard deviation of split frequencies between the two runs for convergence. Of the 20,000 trees sampled in each run, the first 25% trees (i.e., 5000) were discarded as burn-in and the remaining trees were used to construct a 50% majority rule consensus tree. The harmonic mean of likelihoods was estimated for post burn-in trees using the *sump* command in MrBayes.

### Phylogenetic reconstruction of planthopper host data

Phylogenetic analyses of the planthopper host data set were conducted using ML and BI methods. Modeltest 3.7 [39] results using the AIC test [40] indicated that the GTR + I + G model was the best-fitting model for each of the five gene partitions. Partitioned ML analyses were conducted on the planthopper host data using GARLI, with each gene partition set to its optimal model, with these models unlinked and employing their own rates. Ten independent search replicates were run, with each replicate run for 100,000 generations. Bootstrap support values for nodes on the ML topology were computed with GARLI by generating 100 replicates. Unpartitioned ML analyses were also conducted on the planthopper host data using GARLI, under the procedures described above, but with one GTR + I + G model applied to the complete data set.

A mixed-model Bayesian analysis of the planthopper host data was conducted using MrBayes 3.1.2 on the CIPRES Gateway [43]. This analysis was run for 20 million generations, with each partition set to the GTR + I + G model and model parameters unlinked and estimated independently across partitions. Two independent runs were performed, each with four chains, uninformative priors, and trees sampled at intervals of 1000 generations. Stationarity was determined as described above for the BI analysis of the bacterial data set. Of the 20,000 trees sampled in each run, the first 25% trees (i.e., 5000) were discarded as burn-in and the remaining trees were used to construct a 50% majority rule consensus tree. The harmonic mean of likelihoods was estimated for post burn-in trees using the *sump* command in MrBayes.

### Tests of Codiversification of endosymbionts and planthopper hosts

In order to perform statistical tests of codiversification of each of the endosymbionts (the Betaproteobacterial endosymbiont and *Sulcia*) with the planthopper hosts, endosymbiont and host topologies that contain only corresponding sequences (e.g., no outgroup bacterial sequences) were required. Therefore, the following additional analyses were conducted: 1) ML and BI analysis of only the planthopper Betaproteobacterial endosymbiont data, 2) ML and BI analysis of only the planthopper *Sulcia* data, 3) ML and BI analysis of only the host planthopper data for which *Sulcia* was obtained (i.e., *Sulcia* was not successful sequenced for all screened planthoppers), and 4) ML and BI analysis of only the Betaproteobacterial data from taxa for which *Sulcia* was also obtained.

Separate Modeltest results obtained for the planthopper Betaproteobacterial data set and the *Sulcia* data set indicated that the GTR + I + G model was the best-fitting model for each of these data sets. The model parameters specified for each data set were used to calculate pairwise distances of planthopper Betaproteobacterial sequences and *Sulcia* sequences. These additional ML and BI analyses were conducted as described above for the complete bacterial data set and host planthopper data set, respectively (e.g., using the previously described search parameters, number of replicates/generations, etc.). Unpartitioned ML analyses of the subset of the host planthopper data were also conducted with GARLI as described above for the complete host planthopper data set.

Two methods were employed to test codiversification of the endosymbionts with the planthopper hosts and with each other. Topology-based randomization tests were performed using TreeMap [45]. In this test, 1000 randomized endosymbiont trees were mapped onto the host tree (performed separately for the Betaproteobacterial endosymbiont + planthopper hosts, *Sulcia* + planthopper hosts, and *Sulcia* + Betaproteobacterial endosymbiont, respectively), and the number of random codivergences is used to estimate the probability that the observed number of codivergences is due to chance alone. Path-length distance tests were performed with ParaFit [46] using the wrapper program CopyCat [47]. This test generates distance matrices from input endosymbiont and host trees, and tests the global null hypothesis that the two trees are similar due to chance alone [47,48]. Because partitioned versus unpartitioned ML analyses of the host planthopper data yielded somewhat different topologies, TreeMap and ParaFit tests were conducted for both partitioned and unpartitioned ML host trees.

## Competing interests

The authors declare that they have no competing interests.

## Authors’ contributions

JU conceived of the design of the study, generated the endosymbiont and host DNA sequences, performed the phylogenetic analyses, and drafted the manuscript. JC participated in the design of the study and the sequence alignment, and helped to draft the manuscript. Both authors read and approved the final manuscript.

## Availability of supporting data

Alignments of the *Vidania*, *Sulcia*, and planthopper host data, respectively, have been included as Additional files 1, 2, 3.

## Supplementary Material

Additional file 1 **Nucleotide alignment of ***Vidania ***16S data.** Click here for file

Additional file 2 **Nucleotide alignment of ***Sulcia ***16S data.** Click here for file

Additional file 3 Nucleotide alignment of planthopper host 18S, 28S, H3, Wg, COI data. Click here for file
